# Cholinergic Potentiation Improves Perceptual-Cognitive Training of Healthy Young Adults in Three Dimensional Multiple Object Tracking

**DOI:** 10.3389/fnhum.2017.00128

**Published:** 2017-03-21

**Authors:** Mira Chamoun, Frédéric Huppé-Gourgues, Isabelle Legault, Pedro Rosa-Neto, Daniela Dumbrava, Jocelyn Faubert, Elvire Vaucher

**Affiliations:** ^1^Laboratoire de Neurobiologie de la Cognition Visuelle, École d’optométrie, Université de MontréalMontréal, QC, Canada; ^2^Laboratoire de Psychophysique et de Perception Visuelle, École d’optométrie, Université de MontréalMontréal, QC, Canada; ^3^McGill Centre for Studies in AgingDouglas Research Institute, McGill University, Montréal, QC, Canada; ^4^Laboratoire des Neurosciences de la Vision, École d’optométrie, Université de MontréalMontréal, QC, Canada

**Keywords:** 3D-multiple object tracking/NeuroTracker, acetylcholine, acetylcholinesterase inhibitor, attention, cognitive enhancer, donepezil, sensory training, visual learning

## Abstract

A large body of literature supports cognitive enhancement as an effect of cholinergic potentiation. However, it remains elusive whether pharmacological manipulations of cholinergic neurotransmission enhance complex visual processing in healthy individuals. To test this hypothesis, we randomly administered either the cholinergic transmission enhancer donepezil (DPZ; 5 mg P.O.) or placebo (lactose) to young adults (*n* = 17) 3 h before each session of the three-dimensional (3D) multiple object tracking (3D-MOT) task. This multi-focal attention task evaluates perceptual-cognitive learning over five sessions conducted 7 days apart. A significant amount of learning was observed in the DPZ group but not the placebo group in the fourth session. In the fifth session, this learning effect was observed in both groups. Furthermore, preliminary results for a subgroup of participants (*n* = 9) 4–14 months later suggested the cholinergic enhancement effect was long lasting. On the other hand, DPZ had no effect on basic visual processing as measured by a motion and orientation discrimination task performed as an independent one-time, pre-post drug study without placebo control (*n* = 10). The results support the construct that cholinergic enhancement facilitates the encoding of a highly demanding perceptual-cognitive task although there were no significant drug effects on the performance levels compared to placebo.

## Introduction

Sensory training has recently emerged as a promising field in the improvement of perception and cognition (Seitz, 2010). The enhancement of perception and cognition would alleviate sensory shift due to aging or sensory diseases or simply improve daily and sport-related performance. Various training procedures, such as the repetitive presentation of a specific set of stimuli, have been proposed for computer, tablet or three-dimensional (3D) environment (Faubert and Sidebottom, 2012; Kim et al., 2015). Moreover, recent evidence shows that stimulation of the neuromodulatory brain systems may potentiate perceptual learning (long-term improvement of perception induced by sensory training; Rokem and Silver, 2010; Kang et al., 2015).

It has been shown that the cholinergic system improves the efficiency of cortical processing of visual stimuli. Acetylcholine (ACh) optimizes the gain of pyramidal cells in response to sensory stimuli and reduces sensory noise in humans (Moran et al., 2013). This contributes to visual attention and learning processes (Yu and Dayan, 2002; Sarter et al., 2005; Hasselmo, 2006; Herrero et al., 2008). This also increases both sensory precision and the strength of the representation of stimuli. Moreover, in cases of visual training it has been shown that ACh aids cortical processing, improving both its speed and efficiency (Ricciardi et al., 2013). Therefore, combining visual training of a specific stimulus with cholinergic potentiation, namely electrical or pharmacological stimulation of the cholinergic system, induces enhancement of visual perceptual learning (Rokem and Silver, 2010; Beer et al., 2013; Kang et al., 2014a,b).

Animal studies investigating electrical stimulation of cholinergic neurons paired with visual stimulation demonstrate a strong effect on cortical responses in the primary visual cortex (V1), cortical plasticity and visual performance (Goard and Dan, 2009; Kang and Vaucher, 2009; Bhattacharyya et al., 2013; Kang et al., 2014a). However, there are certain issues with implementing this electrical stimulation technique in a human clinical setting. A worthwhile alternative to this cholinergic enhancement is the use of acetylcholinesterase inhibitors (AChEIs), drugs used in Alzheimer’s disease to block the breakdown of ACh at the synapse and prolong its action. Accordingly, AChEIs improve performance of people in visual attention tasks (Demeter and Sarter, 2013) and behavioral tasks that require voluntary attention (Bentley et al., 2004). In rats, donepezil (DPZ), the AChEI most universally used for the clinical treatment of Alzheimer’s disease, has been demonstrated to increase the amplitude of visually evoked potentials when administered during a visual training period (Chamoun et al., 2016). DPZ also improves performance in diverse cognitive behavioral tasks (Wise et al., 2007; Cutuli et al., 2008; Soma et al., 2013). Pharmacological therapy with AChEIs combined with a behavioral intervention is therefore a potentially interesting therapeutic approach to enhancing perceptual-cognitive visual performance in humans.

Therefore, we used the 3D-multiple object tracking (3D-MOT) task to evaluate the effect of cholinergic potentiation on the perceptual-cognitive capacity of healthy young adults. In the 3D-MOT test, tracking of several targets among distractors is done in a 3D environment to increase stereoscopy and demands on attention tracking (Parsons et al., 2016). 3D-MOT thus entails multi-focal attention and requires a high level of processing (Pylyshyn and Storm, 1988; Cavanagh and Alvarez, 2005; Faubert and Sidebottom, 2012; Legault and Faubert, 2012) which encompasses the capacity of the brain to ignore distractors and noise. The task is performed weekly over 5 weeks (a 30-min session once a week). A significant increase in tracking performance is usually measured during the fifth session. 3D-MOT is usually used to improve vision in athletes (Faubert, 2013) and aging persons (Legault and Faubert, 2012; Legault et al., 2013). Since 3D-MOT requires a high attentional load and induces perceptual and cognitive learning, it falls into the range of possible cholinergic involvement and thus should be sensitive to cholinergic potentiation.

We therefore administered DPZ during 3D-MOT perceptual-cognitive training and calculated speed thresholds for tracking moving balls. Moreover, the acute effect of DPZ on basic visual processing was further evaluated with orientation and visual motion discrimination (Hutchinson and Ledgeway, 2006; Allard and Faubert, 2013). We demonstrated that the tracking ability was improved at an earlier time point in healthy young adults taking DPZ, which could be related to attention and efficiency of cortical processing. Moreover, preliminary data suggest a long-lasting effect on performance. This study supports the possibility of using DPZ to improve training of perceptual-cognitive function in humans.

## Materials and Methods

### General Methods

#### Participants

Twenty healthy young adults participated in the study. Two participants were excluded due to a time conflict, and one was excluded due to unsatisfactory baseline performance. The participants (10 men, 7 women; age: 23 ± 1 years; body mass index, BMI: 23 ± 1 kg/m^2^, mean ± SEM, see Table 1) were randomly assigned to either the DPZ (*n* = 9) or placebo (lactose pills, *n* = 8) group. Only some participants were available to perform long-term 3D-MOT task testing (Table 1). Ten participants were independently tested with motion or orientation discrimination tasks (more than 6 months after the 3D-MOT task) using a cross-over design experiment (see Table 1 and below). The sample size (*n* = 10 per group) was determined based on previous studies on the 3D-MOT task (Legault et al., 2013) and visual perception tasks (Allard and Faubert, 2013).

**Table 1:** **Demographic data: participant characteristics and involvement in the three-dimensional (3D)-MOT task and basic visual discrimination tasks**.

Participant	*n*	Age years	Height cm	Weight kg	BMI kg/m^2^
MOT	17	23 ± 1 (20–31)	173 ± 3 (157–193)	69 ± 3 (47–95)	22 ± 1 (19–26)
DPZ group	9	22 ± 1 (20–26)	176 ± 3 (167–193)	71 ± 4 (56–90)	22 ± 1 (19–26)
Placebo	8	24 ± 1 (20–31)	169 ± 4 (157–192)	67 ± 6 (47–95)	23 ± 1 (19–25)
MOT, long-term	9	24 ± 1 (20–31)	173 ± 3 (159–193)	70 ± 4 (50–90)	23 ± 1 (19–25)
Visual discrimination	10	23 ± 1 (20–27)	174 ± 2 (167–193)	69 ± 3 (68–90)	23 ± 1 (19–25)

*Values represent the mean ± SEM (range) of participants in the MOT task, long-term MOT testing and basic motion and orientation discrimination tasks (Visual discrimination). Only a subset of participants could participate in the long-term MOT retesting and visual discrimination task. BMI, body mass index; MOT, multiple object tracking*.

All participants were naive to the purpose of the experiment and met the inclusion and exclusion criteria (Table 2): normal or corrected-to-normal vision, no history of neurological, psychiatric or toxicological problems, no history of smoking, etc. BMI measurements had to be 17–26 kg/m^2^ in order to ascertain a similar distribution of the drug across subjects. A standard clinical and neurological examination, a stereoacuity test and an ECG recording were performed before the beginning of the experiment. All participants completed a written informed consent form prior to the beginning of the experiment. All data were collected and kept secured in the laboratory of Drs. Vaucher and Faubert at the School of Optometry. The participants were enrolled by the student researcher, MC, and their random allocation sequence was carried out by EV by assigning drug/placebo in numbered containers. Numbers were assigned to participants in order of participation. Subjects received financial compensation to cover travel expenses and time spent participating in the experiment. The procedures were in accordance with the Helsinki Declaration of 2013 and the ethical standards of the Comité d’éthique de la recherche en santé, Université de Montréal, approval #12-084-CERES-P. The study was registered on ClinicalTrials.gov (NCT01738295).

**Table 2:** **Inclusion and exclusion criteria**.

Inclusion criteria	Exclusion criteria
Aged 20–35	Previous MOT task participant
Good health	Attention deficit
Body mass index between 17 and 26	Smoker
No visual impairment or ocular pathology not corrected by glasses or contact lenses	Pregnant, breast feeding or planning a pregnancy
Good 3D vision	Lactose intolerance

#### Donepezil Pharmacological Enhancement

DPZ is a reversible, non-competitive, highly selective AChEI with a half-life of 80 h and a peak plasma level of 4.1 ± 1.5 h after intake (Rogers and Friedhoff, 1998). The 5 mg dose was selected because it is the lowest prescribed dose which induces beneficial cognitive effects with very low adverse reaction incidence (Prvulovic and Schneider, 2014). This dose has been shown to be effective in improving visual attention and neural plasticity in young adults (Rokem and Silver, 2010; Rokem et al., 2010). Three hours before each session, subjects were administered one capsule containing either 5 mg DPZ (ARICEPT^®^, Pfizer, Canada) or lactose placebo with water (Rokem and Silver, 2010). The experimenter and subjects were naive to the experimental conditions. We used commercially available DPZ tablets (ARICEPT^®^, Pfizer, Canada) but this company has no financial interest in this study.

### Experiment 1: 3D-Multiple Object Tracking

The goal of this experiment was to determine if acute administration of DPZ at each weekly session of the 3D-MOT task over five consecutive sessions could improve the tracking of four objects either in terms of performance threshold or learning rate. An additional test of the 3D-MOT task was carried out with the available participants 4–14 months after the end of training without drug intake (*n* = 5, DPZ group; *n* = 4, control group, nine participants from the original 17, see Table 1) to assess the preliminary results of the long-lasting effect of cholinergic enhancement on this perceptual-cognitive task.

#### Experimental Design

All subjects were tested on the 3D-MOT task once a week for five consecutive weeks. The first week was used as a baseline measurement and was done without administering the drug or placebo. This was a double-blind placebo controlled intervention. The study drug or placebo was administered P.O. 3 h before the testing for the next 4 weeks.

#### Procedure

**Figure 1:**
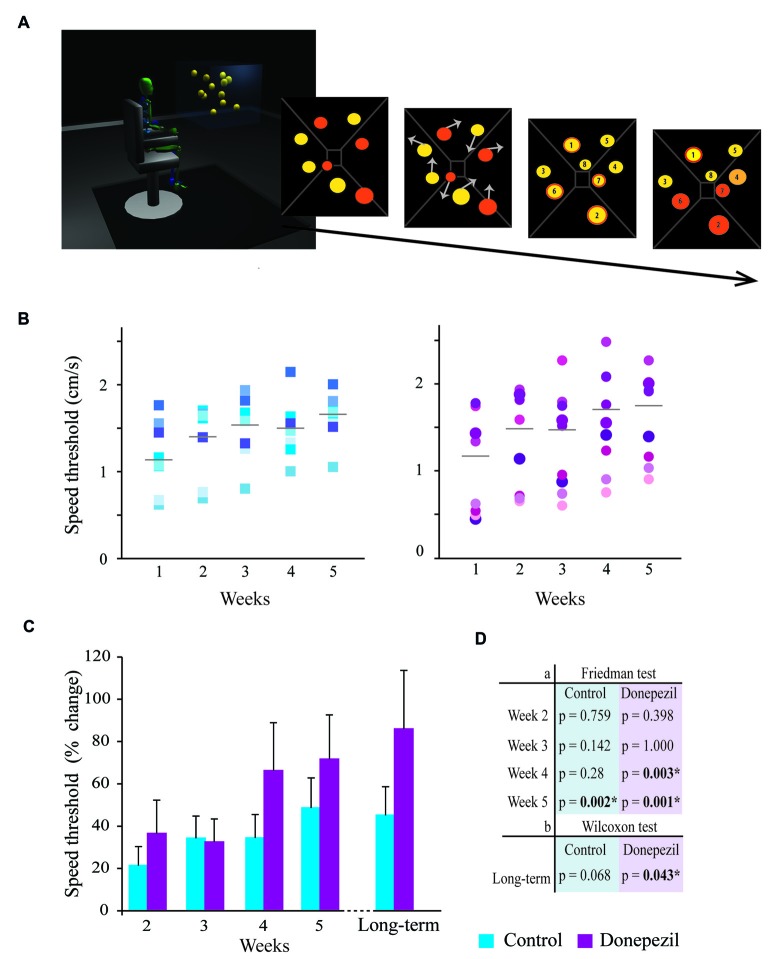
**Three-dimensional (3D)-multiple object tracking (3D-MOT) task: comparison of tracking performance in the donepezil (DPZ) and placebo group. (A)** Example of the 3D-MOT: eight yellow spheres are randomly positioned in a virtual 3D environment; four randomly selected spheres turn orange for identification of the spheres to track (targets); then all spheres turn back to yellow and move following a random linear trajectory (arrows represent initial movement) at a defined speed; the spheres stop, are numbered and the participant identifies the targets (orange border); finally, the targets turn orange for correct feedback; the targets turn yellow with a red border for a wrong answer and the targets turn light orange for a right answer not identified. The trial is then repeated, changing the speed of the movement of the spheres using a 1-up-1-down staircase procedure. The speed threshold for which the subjects are able to track balls is calculated from the mean of the last four reversals of the staircase. **(B)** Tracking performance in terms of speed threshold (cm/s) for each participant every testing week (Weeks 1–5) in the control group (blue-shaded squares) and DPZ group (purple-shaded circles). The mean of the speed threshold (gray bar) is given. **(C)** Speed threshold (percent change from baseline) for tracking performance of subjects every testing week and during long-term testing (4–14 months after the initial training) for the control group (in blue) and the DPZ group (in purple). **(D)** The significance table represents (a) the statistical comparison of the mean speed threshold for each group using Friedman’s test with Bonferroni correction (upper panel). Note that the DPZ group significantly improved their performance (significant difference in speed threshold compared to baseline value) at Weeks 4 and 5, while the control group only reached this level of improvement at Week 5; (b) the statistical comparison of the mean speed threshold of long-term testing to baseline values using the Wilcoxon test. There was a significant sustained improvement in the speed threshold in the DPZ group but not in the control group. *Significantly different compared to baseline (week 1), *p* < 0.05.

The task consisted of eight yellow spheres projected in the Cave Automatic Virtual Environment (CAVE). The CAVE is a fully immersive virtual environment consisting of an 8 × 8 × 8 foot room that includes three canvas walls (one frontal and two lateral walls) and an epoxy floor that serve as the surface for image projection (Figure 1A). Each participant sat 177 cm from the central wall of the CAVE with eye height set at 160 cm from the ground. They were asked to wear stereoscopic goggles to visualize the 3D-environment and to fixate on a point located straight ahead of them. Four of the balls turned orange for identification. Then all spheres turned back to yellow and followed a linear trajectory with a selected speed. The spheres moved in a 3D-volume space, sometimes bouncing off of or occluding one another or bouncing off the virtual wall (Figure 1A). This movement activity lasted 8 s and then stopped. Next, participants had to identify the target spheres. They received feedback as to their correct or incorrect response. The next speed was determined using a 1-up-1-down staircase procedure (Levitt, 1971; Legault et al., 2013). The staircase was interrupted after eight reversals. The speed thresholds were established from the mean of the last four staircase reversals. Each testing session consisted of three repetitions of the same block, each of which lasted approximately 10 min.

#### Experimental Setting

As previously described (Legault et al., 2013), four high-resolution projectors were synchronized and the image was updated in real-time to maintain the observer’s true viewing perspective (i.e., no false parallax). A magnetic motion tracker system (Flock-of-Birds) was used to measure head position, which was used to correct the viewing perspective of the observer in real-time. The CAVE was under the control of a SGI ONYX 3200 computer (two Infinite Reality 2 graphic cards), which produced a stereoscopic environment. The stereoscopy was generated with Crystal Eyes 2 active shutter glasses synchronized at 96 Hz (48 Hz per eye). Before testing, subjects were familiarized with the virtual environment and stimuli.

### Experiment 2: Orientation and Motion Visual Perception

The goal of this experiment was to test whether DPZ alters basic visual processing, tested in a motion and orientation discrimination task. This task was tested independently from the 3D-MOT task (at least 6 months after the last drug intake).

#### Experimental Design

Ten healthy young adults (6 men, 4 women; age: 24 ± 1 years; BMI: 23 ± 1 kg/m^2^; mean ± SEM, nine participants from the original 17 and 1 new participant; see Table 1) took part in a pre-post drug study without placebo control. All subjects were tested on the orientation and motion visual perception task once before and 3 h after receiving DPZ at the peak DPZ plasma concentration. However, the subjects were told that they could receive either placebo or DPZ.

#### Procedure

**Figure 2:**
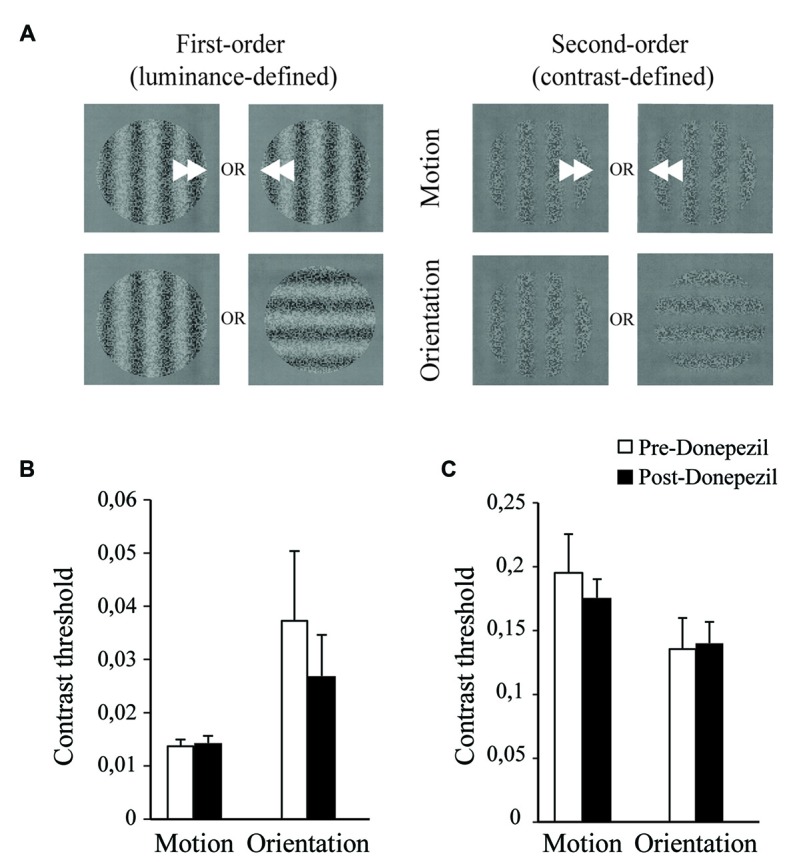
**Motion and orientation discrimination: acute effect of a one-time administration of DPZ. (A)** Representation of the basic visual perception tasks of motion (upper panel) and orientation (lower panel) discrimination of the stimulus. The stimulus is a sine-wave grating of 0.3 cycles per degree modulated by discrete variations of luminance, first-order luminance defined stimulus (left panel) or contrast, second-order contrast defined stimulus (right panel). Average Michelson contrast thresholds are shown for luminance-defined stimulus **(B)** for the discrimination of motion (left) or orientation (right) before (open bar) and 3 h after (black bar) DPZ intake, and for the contrast-defined stimulus **(C)** for the discrimination of motion (left) or orientation (right) before (open bar) and 3 h after (black bar) DPZ intake. For both the motion and the orientation discrimination tasks, neither the mean luminance modulation threshold nor the mean contrast modulation thresholds reached by the participants were altered by the intake of DPZ (Wilcoxon test, *p* > 0.05). Error bars represent SEM values.

The task was performed as previously designed and validated (Allard and Faubert, 2013). The observer was positioned in a dark room 114 cm from the display and familiarized with the task. A trial consisted of identifying the motion or the orientation of a sine-wave grating of 0.3 CPD, by pushing arrow keys on a keyboard (Figure 2A). A feedback sound indicated whether the response was correct or incorrect. Motion stimuli were composed of either a luminance or a contrast modulation of a sine-wave grating drifting in a random direction (either left or right). All modulations were vertically oriented. First-order motion stimulus (luminance modulation) drifted at 15 Hz and the second-order motion stimulus (contrast modulation) at 2 Hz. The orientation task was a horizontal or vertical display of the sine-wave grating. The contrast or luminance modulation was controlled by a 2-down-1-up staircase procedure (Levitt, 1971; Allard and Faubert, 2008a) and the luminance or contrast threshold obtained for the stimulus discrimination was estimated for the last six reversals of the staircase. The testing consisted of three repetitions of four blocks of trials; each block presented one of the four conditions (first- and second-order motion and first-and second-order orientation) in a randomized manner. The testing duration was approximately 30 min.

#### Experimental Setting

Stimuli were presented on a gamma-linearized 22″ Formac ProNitron 22800 CRT monitor with a mean luminance of 42 cd/m^2^ and a refresh rate set at 120 Hz. The spatial window was circular with a diameter of 4°. The presentation time was 500 ms. The noisy-bit method (Allard and Faubert, 2008b) was implemented to improve the screen luminance resolution and make it perceptually equivalent to a continuous resolution. The noise was dynamic, spatiotemporally extended and presented over the entire screen and visible at all times. The noise was binary and elements were 2 × 2 pixels wide (i.e., 0.028 × 0.028°).

### Statistical Analysis

For the motion and orientation visual perception tests, mean luminance/contrast threshold were analyzed using a Wilcoxon test between the testings performed before and after the intake of DPZ. The progression of the speed thresholds in the MOT task was analyzed in each group separately using a Friedman test with Bonferroni correction (*p* < 0.01) for multiple comparisons of speed threshold values between the baseline (Week 1) and the training weeks (Weeks 2–5). An additional comparison was conducted to examine the difference in performance between both groups (control and DPZ) using the Kruskal-Wallis test to compare speed thresholds at each time point (Weeks 1–5). Long-term testing of the 3D-MOT speed threshold was compared to the speed threshold of the first week (baseline) from the returning participants using a Wilcoxon test. Statistical analyses were performed using SPSS 17.0 (SPSS Inc., Chicago, IL, USA).

## Results

### Donepezil Effect on 3D-MOT Training

The 3D-MOT task is a training task conducted over five consecutive weeks during which participants gradually improve their tracking performance. The subjects taking DPZ were able to successfully track four balls with significantly greater speed as early as the fourth session (*p* = 0.003) compared to their baseline values (measured during the first session). This result was maintained in the fifth session (*p* = 0.001; Figure 1). In contrast, the control group only showed a significant improvement in speed threshold during the fifth training session (*p* = 0.002; Figure 1). However, the speed threshold for which the subjects were able to track balls was not significantly different between the DPZ and control group at any time point (Kruskal-Wallis test, *p* > 0.05) although percentage of change from baseline was 20%–50% higher for the DPZ group compared to the placebo group for each session after week 3.

### Donepezil Effect on the 3D-MOT Task is Long-Lasting

Performance of the 3D-MOT task was also measured 4–14 months following the last testing session depending on participant availability (Figures 1C,D). The average amount of time between the last training session and the retesting session was not significantly different between the two groups (Kruskal-Wallis, *p* = 0.711). A significant increase in the speed threshold (86%) obtained by the initial DPZ group was observed at this late time point compared to baseline values (Week 1; Wilcoxon test, *p* = 0.043). The control group maintained good tracking skills but the increase in speed threshold (45%) compared to baseline values was not significant at that point (Wilcoxon test, *p* = 0.068).

### Acute Administration of Donepezil does Not Affect First- and Second-Order Stimuli Discrimination

To evaluate the acute effect of DPZ on basic visual processing, motion and orientation discrimination abilities were tested before and 3 h after DPZ administration. The motion discrimination threshold was not affected by DPZ either in terms of first-order stimuli (*p* = 0.878) or second-order stimuli (*p* = 0.878; Figures 2B,C). Additionally, orientation discrimination was not affected by DPZ for either first-order stimuli (*p* = 0.515) or second-order stimuli (*p* = 0.386; Figures 2B,C). Together these results suggest that acute cholinergic potentiation does not influence basic visual processing of orientation and motion. The fact that no difference was observed for both levels of processing might be explained by the lack of repetitive training/DPZ administration that could not induce an optimal recruitment of cholinergic neurons or by an optimal performance of the young subjects, which might not be further increased.

## Discussion

This study demonstrates that an increase in cholinergic transmission by the AChEIs, DPZ, has no significant effect on the tracking skills in the 3D-MOT task compared to placebo but may improve the learning in this 5-week perceptual-cognitive training procedure (1 session/week). In addition, preliminary results suggest that the training effect was maintained after 4–14 months in the DPZ group but not in the control group. This long-term result is preliminary, due to the small sample of returning participants. The basic visual discrimination of first- or second-order stimuli did not seem to be affected by cholinergic potentiation as measured in an additional one-time, pre-post drug study without placebo control. Together, these results suggest the role of the cholinergic system in fine-tuning complex visual perceptual-cognitive processing. The discussion emphasizes how this DPZ effect might be due to the role of ACh in attentional processes, optimization of visual efficiency (refining of cortical circuitry and increase in sustained neural firing), improvement of the signal/noise ratio, repetitive training, or all these effects combined.

### Cholinergic Enhancement Improves Training but Not Performance in a Multi-Focal Attention Task in Healthy Young Adults

The present results showed that DPZ facilitates the learning in a 5-week multi-focal attention 3D-MOT training procedure. The overall maximum performance measured at the fifth session was however not significantly improved by DPZ administration, suggesting a learning effect rather than an improvement of performance *per se*. This learning effect could be due to a potentiation of the attentional capacities by increased brain concentrations of ACh. This result is in line with previous studies showing that ACh is involved in visual attention processes in primates (Voytko et al., 1994; Bentley et al., 2004; Herrero et al., 2008) and that DPZ potentiates performance in attentional tasks (Rokem et al., 2010). As well, the cholinergic enhancement by physostigmine (another cholinesterase inhibitor) potentiates selective attention by improving selectivity to the relevant stimuli (Furey et al., 2000; Ricciardi et al., 2009). In the 3D-MOT task, both selective and divided attention are involved (Cavanagh and Alvarez, 2005; Doran and Hoffman, 2010) and could be potentiated by a higher ACh concentration.

The learning effect of DPZ on 3D-MOT training could also be due to a refinement of the cortical circuitry sustaining the accomplishment of the task. 3D-MOT training increases by itself attention, working memory and visual information processing speed. Moreover, 3D-MOT training induces changes in resting-state functional brain imaging and electroencephalography, more specifically a decrease in the theta, alpha and gamma frequencies in the frontal lobe and increased gamma frequency over the occipital lobes (Parsons et al., 2016). It has been established that cholinergic activity during top-down attention processes also influences interactions between the different brain areas (Golmayo et al., 2003; Nelson et al., 2005). For instance, the improvement in working memory performance following a steady-state infusion of the AChEI physostigmine was accompanied by increased activity in the visual cortex associated with perceptual processing, whereas activity in the prefrontal cortex was decreased, suggesting a reduction of attentional load to perform the same task (Furey et al., 2000). ACh also plays a role in reducing the spatial spread of visual responses in early visual cortex tested in fMRI (Silver et al., 2008). Given that 3D-MOT is a task that requires higher levels of processing (Faubert and Sidebottom, 2012) and involves multiple brain areas (Culham et al., 1998; Jovicich et al., 2001), coupling this task with cholinergic enhancement benefits from the role of the cholinergic system in increasing the activation of the sensory cortex and the refinement of processing. Improvement of the 3D-MOT performance by regional activation has also been shown by transcranial current brain stimulation of certain structures (anterior intraparietal sulcus, for example) in the visual pathway (Blumberg et al., 2015).

An additional role of cholinergic potentiation in faster improvement of tracking skill of participants is the possible modulation of the signal-to-noise ratio, thus making relevant stimuli sharper. Some studies demonstrate that ACh can increase the processing of relevant stimuli and suppress the processing of irrelevant input in a top-down attention task. Human studies show that ACh allows a clearer perceptual representation of the target by reducing background noise (Furey et al., 2000; Ricciardi et al., 2009). In rats, cholinergic enhancement is associated with an improvement in the signal-to-noise ratio therefore enhancing the rat’s response to a visual stimulus (Kang et al., 2014a). Therefore, administering DPZ could have had an effect in reducing the noise level, allowing the participants to have a clearer representation of the targets among the distractors.

3D-MOT performance usually improves during training because of specific perceptual-cognitive learning activity (Makovski et al., 2008). Given the role of ACh in learning, pairing visual training with ACh enhancement may modulate the tuning of the neurons for the trained stimuli (Rokem and Silver, 2010). In fact, studies in rats and humans have shown the role of cholinergic enhancement of visual training on the potentiation of visual capacities such as visual cortical responsiveness (Kang et al., 2014a; Chamoun et al., 2016), contrast sensitivity (Soma et al., 2013; Boucart et al., 2015), motion direction discrimination (Rokem and Silver, 2010) and texture discrimination (Beer et al., 2013). In addition, given the role of the cholinergic system in attention and the role of attention in perceptual learning (Ahissar and Hochstein, 1993), pairing visual training with ACh enhancement may fine-tune the allocation of attention in this multi-focal attention task. Therefore, perceptual-cognitive learning combined with DPZ administration might optimize multi-focal tracking skills. The lack of effect of DPZ on the absolute performance value could be indicative of a ceiling effect due to high performance and cholinergic activity in young healthy subjects preventing further improvement of performance.

### The Cholinergic Enhancement Effect Is Long-Lasting

The present study suggests that cholinergic enhancement has a long-lasting effect on tracking performance. However, the long-term effect was tested on a smaller sample of participants (*n* = 9) and further studies need to be conducted to verify such an effect. This lasting effect would correspond with previous studies showing that an improvement in visual learning after training on a motion task lasts for months after the training (Ball and Sekuler, 1982). However, the cholinergic enhancement doubled the amount of learning compared to the control subjects at this time point, suggesting that the cholinergic effect on visual learning and consolidation of the learning or recall mechanism (Zaninotto et al., 2009) amplifies retention of performance skills. Likewise, in a previous study, participants were trained on a texture discrimination task and were given an ACh agonist (nicotine) at the end of the training session, indicating an effect of ACh on consolidation processes (Beer et al., 2013). The present results indicate that an effect of AChEI on the consolidation of learning of a trained task is possible. In animal models, long-term potentiation-like effects of the cholinergic system have been observed on visual evoked potentials after basal forebrain stimulation (Kang et al., 2014a), suggesting the formation of memory traces in the visual cortex. Together these findings suggest a role of cholinergic enhancement in the encoding and long-term perceptual-cognitive learning processes following visual training.

### Limitation of the Study

This study was conducted with a relatively small sample of participants, reducing the power of the statistical analysis. While this sample size is regularly used in corresponding studies, we obviously need a greater sample size to more thoroughly assess possible effects of DPZ on the performance level in the 3D-MOT task. Also, the long-term data are for a smaller number of returning participants and should be further confirmed. Further experiments will also be required to isolate the transfer effects of this cholinergic potentiation on attention or perception. However, it is known that 3D-MOT training over 5 weeks can enhance cognition and changes in resting-state neuroelectric brain function (Parsons et al., 2016).

## Conclusion

In conclusion, repetitive DPZ administration during perceptual-cognitive training led to an earlier improvement of tracking ability and to a potential long-lasting effect. We believe that this effect is due to a combination of the effect of ACh on attention, perception and fine tuning of visual processing. These results suggest the importance of boosting the cholinergic system in practicing visual tasks to enhance plastic changes and efficacy of visual processing and memory traces. This effect would help in improving rehabilitation strategies to help visually impaired people recover vision capacity.

## Author Contributions

EV: director of the Laboratory of neurobiology of the visual cognition at the University of Montréal; contributed by designing the project, providing the material, analyzing the data and writing the article. MC: contributed by designing the project, testing the participants, analyzing the data and writing the article. FH-G: contributed by analyzing the data and writing the article. IL: contributed by designing the project and analyzing the data. JF: contributed by designing the project, providing the 3D-MOT and the Orientation and Motion Visual Perception techniques and equipment, analyzing the data and writing the article. PR-N: contributed by designing the project, providing the donepezil pills and writing the article. DD: contributed by performing the medical examination of the participants.

## Funding

Grant sponsor: Canadian Institutes of Health Research; Grant number: MOP-111003, EV. Natural Sciences and Engineering Research Council of Canada; Grant number: 238835-2011, EV. MC received financial support from the School of Optometry, Université de Montréal. We would like to thank the Centre for Interdisciplinary Research in Rehabilitation of Greater Montreal for financial resource support.

## Conflict of Interest Statement

JF is director of the Visual Psychophysics and Perception Laboratory at the University of Montreal and he is the Chief Science Officer of CogniSens Athletics Inc. who produce the commercial version of the 3D-MOT program (NeuroTracker) used in this study. In this capacity, he holds shares in the company. The other authors declare that the research was conducted in the absence of any commercial or financial relationships that could be construed as a potential conflict of interest.

## References

[B1] Ahissar M. Hochstein S. (1993). Attentional control of early perceptual learning. Proc. Natl. Acad. Sci. U S A 90, 5718–5722. 10.1073/pnas.90.12.5718 8516322 PMC46793

[B2] Allard R. Faubert J. (2008a). First- and second-order motion mechanisms are distinct at low but common at high temporal frequencies. J. Vis. 8, 12, 1–17. 10.1167/8.2.12 18318638

[B3] Allard R. Faubert J. (2008b). The noisy-bit method for digital displays: converting a 256 luminance resolution into a continuous resolution. Behav. Res. Methods 40, 735–743. 10.3758/brm.40.3.735 18697669

[B4] Allard R. Faubert J. (2013). No second-order motion system sensitive to high temporal frequencies. J. Vis. 13:4. 10.1167/13.5.4 23559594

[B5] Ball K. Sekuler R. (1982). A specific and enduring improvement in visual motion discrimination. Science 218, 697–698. 10.1126/science.7134968 7134968

[B6] Beer A. L. Vartak D. Greenlee M. W. (2013). Nicotine facilitates memory consolidation in perceptual learning. Neuropharmacology 64, 443–451. 10.1016/j.neuropharm.2012.06.019 22749926

[B7] Bentley P. Husain M. Dolan R. J. (2004). Effects of cholinergic enhancement on visual stimulation, spatial attention, and spatial working memory. Neuron 41, 969–982. 10.1016/s0896-6273(04)00145-x 15046728

[B8] Bhattacharyya A. Veit J. Kretz R. Bondar I. Rainer G. (2013). Basal forebrain activation controls contrast sensitivity in primary visual cortex. BMC Neurosci. 14:55. 10.1186/1471-2202-14-55 23679191 PMC3662585

[B9] Blumberg E. J. Peterson M. S. Parasuraman R. (2015). Enhancing multiple object tracking performance with noninvasive brain stimulation: a causal role for the anterior intraparietal sulcus. Front. Syst. Neurosci. 9:3. 10.3389/fnsys.2015.00003 25698943 PMC4318277

[B10] Boucart M. Bubbico G. Szaffarczyk S. Defoort S. Ponchel A. Waucquier N.. (2015). Donepezil increases contrast sensitivity for the detection of objects in scenes. Behav. Brain Res. 292, 443–447. 10.1016/j.bbr.2015.06.037 26162753

[B11] Cavanagh P. Alvarez G. A. (2005). Tracking multiple targets with multifocal attention. Trends Cogn. Sci. 9, 349–354. 10.1016/j.tics.2005.05.009 15953754

[B12] Chamoun M. Groleau M. Bhat M. Vaucher E. (2016). Dose-dependent effect of donepezil administration on long-term enhancement of visually evoked potentials and cholinergic receptor overexpression in rat visual cortex. J. Physiol. Paris 110, 65–74. 10.1016/j.jphysparis.2016.11.010 27913166

[B13] Culham J. C. Brandt S. A. Cavanagh P. Kanwisher N. G. Dale A. M. Tootell R. B. (1998). Cortical fMRI activation produced by attentive tracking of moving targets. J. Neurophysiol. 80, 2657–2670. 9819271 10.1152/jn.1998.80.5.2657

[B14] Cutuli D. Foti F. Mandolesi L. De Bartolo P. Gelfo F. Federico F.. (2008). Cognitive performance of healthy young rats following chronic donepezil administration. Psychopharmacology 197, 661–673. 10.1007/s00213-008-1084-0 18309476

[B15] Demeter E. Sarter M. (2013). Leveraging the cortical cholinergic system to enhance attention. Neuropharmacology 64, 294–304. 10.1016/j.neuropharm.2012.06.060 22796110 PMC3445745

[B16] Doran M. M. Hoffman J. E. (2010). The role of visual attention in multiple object tracking: evidence from ERPs. Atten. Percept. Psychophys. 72, 33–52. 10.3758/APP.72.1.33 20802834 PMC2927139

[B17] Faubert J. (2013). Professional athletes have extraordinary skills for rapidly learning complex and neutral dynamic visual scenes. Sci. Rep. 3:1154. 10.1038/srep01154 23378899 PMC3560394

[B18] Faubert J. Sidebottom L. (2012). Perceptual-cognitive training of athletes. J. Clin. Sport Psychol. 6, 85–102. 10.1123/jcsp.6.1.85

[B19] Furey M. L. Pietrini P. Haxby J. V. (2000). Cholinergic enhancement and increased selectivity of perceptual processing during working memory. Science 290, 2315–2319. 10.1126/science.290.5500.2315 11125148

[B20] Goard M. Dan Y. (2009). Basal forebrain activation enhances cortical coding of natural scenes. Nat. Neurosci. 12, 1444–1449. 10.1038/nn.2402 19801988 PMC3576925

[B21] Golmayo L. Nuñez A. Zaborszky L. (2003). Electrophysiological evidence for the existence of a posterior cortical-prefrontal-basal forebrain circuitry in modulating sensory responses in visual and somatosensory rat cortical areas. Neuroscience 119, 597–609. 10.1016/s0306-4522(03)00031-9 12770572

[B22] Hasselmo M. E. (2006). The role of acetylcholine in learning and memory. Curr. Opin. Neurobiol. 16, 710–715. 10.1016/j.conb.2006.09.002 17011181 PMC2659740

[B23] Herrero J. L. Roberts M. J. Delicato L. S. Gieselmann M. A. Dayan P. Thiele A. (2008). Acetylcholine contributes through muscarinic receptors to attentional modulation in V1. Nature 454, 1110–1114. 10.1038/nature07141 18633352 PMC2666819

[B24] Hutchinson C. V. Ledgeway T. (2006). Sensitivity to spatial and temporal modulations of first-order and second-order motion. Vision Res. 46, 324–335. 10.1016/j.visres.2005.03.002 16360001

[B25] Jovicich J. Peters R. J. Koch C. Braun J. Chang L. Ernst T. (2001). Brain areas specific for attentional load in a motion-tracking task. J. Cogn. Neurosci. 13, 1048–1058. 10.1162/089892901753294347 11784443

[B27] Kang J. I. Groleau M. Dotigny F. Giguere H. Vaucher E. (2014a). Visual training paired with electrical stimulation of the basal forebrain improves orientation-selective visual acuity in the rat. Brain Struct. Funct. 219, 1493–1507. 10.1007/s00429-013-0582-y 23700106

[B28] Kang J. I. Huppe-Gourgues F. Vaucher E. (2014b). Boosting visual cortex function and plasticity with acetylcholine to enhance visual perception. Front. Syst. Neurosci. 8:172. 10.3389/fnsys.2014.00172 25278848 PMC4167004

[B29] Kang J. I. Huppé-Gourgues F. Vaucher E. (2015). Pharmacological mechanisms of cortical enhancement induced by the repetitive pairing of visual/cholinergic stimulation. PLoS One 10:e0141663. 10.1371/journal.pone.0141663 26513575 PMC4626033

[B26] Kang J. I. Vaucher E. (2009). Cholinergic pairing with visual activation results in long-term enhancement of visual evoked potentials. PLoS One 4:e5995. 10.1371/journal.pone.0005995 19543405 PMC2696093

[B30] Kim D. Seitz A. R. Watanabe T. (2015). Visual perceptual learning by operant conditioning training follows rules of contingency. Vis. Cogn. 23, 147–160. 10.1080/13506285.2015.1015663 26028984 PMC4443931

[B31] Legault I. Allard R. Faubert J. (2013). Healthy older observers show equivalent perceptual-cognitive training benefits to young adults for multiple object tracking. Front. Psychol. 4:323. 10.3389/fpsyg.2013.00323 23761025 PMC3674476

[B32] Legault I. Faubert J. (2012). Perceptual-cognitive training improves biological motion perception: evidence for transferability of training in healthy aging. Neuroreport 23, 469–473. 10.1097/WNR.0b013e328353e48a 22495038

[B33] Levitt H. (1971). Transformed up-down methods in psychoacoustics. J. Acoust. Soc. Am. 49, 467–477. 10.1121/1.1912375 5541744

[B34] Makovski T. Vázquez G. A. Jiang Y. V. (2008). Visual learning in multiple-object tracking. PLoS One 3:e2228. 10.1371/journal.pone.0002228 18493599 PMC2375111

[B35] Moran R. J. Campo P. Symmonds M. Stephan K. E. Dolan R. J. Friston K. J. (2013). Free energy, precision and learning: the role of cholinergic neuromodulation. J. Neurosci. 33, 8227–8236. 10.1523/JNEUROSCI.4255-12.2013 23658161 PMC4235126

[B36] Nelson C. L. Sarter M. Bruno J. P. (2005). Prefrontal cortical modulation of acetylcholine release in posterior parietal cortex. Neuroscience 132, 347–359. 10.1016/j.neuroscience.2004.12.007 15802188

[B37] Parsons B. Magill T. Boucher A. Zhang M. Zogbo K. Bérubé S.. (2016). Enhancing cognitive function using perceptual-cognitive training. Clin. EEG Neurosci. 47, 37–47. 10.1177/1550059414563746 25550444

[B38] Prvulovic D. Schneider B. (2014). Pharmacokinetic and pharmacodynamic evaluation of donepezil for the treatment of Alzheimer’s disease. Expert Opin. Drug Metab. Toxicol. 10, 1039–1050. 10.1517/17425255.2014.915028 24785550

[B39] Pylyshyn Z. W. Storm R. W. (1988). Tracking multiple independent targets: evidence for a parallel tracking mechanism. Spat. Vis. 3, 179–197. 10.1163/156856888x00122 3153671

[B40] Ricciardi E. Handjaras G. Bernardi G. Pietrini P. Furey M. L. (2013). Cholinergic enhancement reduces functional connectivity and BOLD variability in visual extrastriate cortex during selective attention. Neuropharmacology 64, 305–313. 10.1016/j.neuropharm.2012.07.003 22906685 PMC3445804

[B41] Ricciardi E. Pietrini P. Schapiro M. B. Rapoport S. I. Furey M. L. (2009). Cholinergic modulation of visual working memory during aging: a parametric PET study. Brain Res. Bull. 79, 322–332. 10.1016/j.brainresbull.2009.01.013 19480991 PMC3264397

[B42] Rogers S. L. Friedhoff L. T. (1998). Pharmacokinetic and pharmacodynamic profile of donepezil HCl following single oral doses. Br. J. Clin. Pharmacol. 46, 1–6. 10.1046/j.1365-2125.1998.0460s1001.x 9839758 PMC1873812

[B44] Rokem A. Landau A. N. Garg D. Prinzmetal W. Silver M. A. (2010). Cholinergic enhancement increases the effects of voluntary attention but does not affect involuntary attention. Neuropsychopharmacology 35, 2538–2544. 10.1038/npp.2010.118 20811340 PMC2978769

[B43] Rokem A. Silver M. A. (2010). Cholinergic enhancement augments magnitude and specificity of visual perceptual learning in healthy humans. Curr. Biol. 20, 1723–1728. 10.1016/j.cub.2010.08.027 20850321 PMC2953574

[B45] Sarter M. Hasselmo M. E. Bruno J. P. Givens B. (2005). Unraveling the attentional functions of cortical cholinergic inputs: interactions between signal-driven and cognitive modulation of signal detection. Brain Res. Rev. 48, 98–111. 10.1016/j.brainresrev.2004.08.006 15708630

[B46] Seitz A. R. (2010). Sensory learning: rapid extraction of meaning from noise. Curr. Biol. 20, R643–R644. 10.1016/j.cub.2010.06.017 20692614

[B47] Silver M. A. Shenhav A. D–Esposito M. (2008). Cholinergic enhancement reduces spatial spread of visual responses in human early visual cortex. Neuron 60, 904–914. 10.1016/j.neuron.2008.09.038 19081383 PMC2640421

[B48] Soma S. Suematsu N. Shimegi S. (2013). Cholinesterase inhibitor, donepezil, improves visual contrast detectability in freely behaving rats. Behav. Brain Res. 256, 362–367. 10.1016/j.bbr.2013.08.022 23994545

[B49] Voytko M. L. Olton D. S. Richardson R. T. Gorman L. K. Tobin J. R. Price D. L. (1994). Basal forebrain lesions in monkeys disrupt attention but not learning and memory. J. Neurosci. 14, 167–186. 8283232 10.1523/JNEUROSCI.14-01-00167.1994 PMC6576852

[B50] Wise L. E. Iredale P. A. Stokes R. J. Lichtman A. H. (2007). Combination of rimonabant and donepezil prolongs spatial memory duration. Neuropsychopharmacology 32, 1805–1812. 10.1038/sj.npp.1301297 17213845

[B51] Yu A. J. Dayan P. (2002). Acetylcholine in cortical inference. Neural Netw. 15, 719–730. 10.1016/s0893-6080(02)00058-8 12371522

[B52] Zaninotto A. L. Bueno O. F. Pradella-Hallinan M. Tufik S. Rusted J. Stough C.. (2009). Acute cognitive effects of donepezil in young, healthy volunteers. Hum. Psychopharmacol. 24, 453–464. 10.1002/hup.1044 19637397

